# Supplementary material for: The Global Prevalence of Anxiety Among Medical Students: A Meta-Analysis
Source: Int J Environ Res Public Health. 2019 Jul 31;16(15):2735. doi: 10.3390/ijerph16152735 (PMC6696211; doi:10.3390/ijerph16152735)
Supplement: Supplementary file 1 [file ijerph-16-02735-s001.pdf]

International Journal of
Environmental Research and Public Health

MDPI

Supplementary Materials

Section/topic	#	Checklist item	Reported on page #
**TITLE**			
Title	1	Identify the report as a systematic review, meta-analysis, or both.	1
**ABSTRACT**			
Structured summary	2	Provide a structured summary including, as applicable: background; objectives; data sources; study eligibility criteria, participants, and interventions; study appraisal and synthesis methods; results; limitations; conclusions and implications of key findings; systematic review registration number.	1
**INTRODUCTION**			
Rationale	3	Describe the rationale for the review in the context of what is already known.	1-2
Objectives	4	Provide an explicit statement of questions being addressed with reference to participants, interventions, comparisons, outcomes, and study design (PICOS).	1-2
**METHODS**			
Protocol and registration	5	Indicate if a review protocol exists, if and where it can be accessed (e.g., Web address), and, if available, provide registration information including registration number.	2

Eligibility criteria	6	Specify study characteristics (e.g., PICOS, length of follow-up) and report characteristics (e.g., years considered, language, publication status) used as criteria for eligibility, giving rationale.	2
Information sources	7	Describe all information sources (e.g., databases with dates of coverage, contact with study authors to identify additional studies) in the search and date last searched.	2
Search	8	Present full electronic search strategy for at least one database, including any limits used, such that it could be repeated.	Appendix 1
Study selection	9	State the process for selecting studies (i.e., screening, eligibility, included in systematic review, and, if applicable, included in the meta-analysis).	2-3
Data collection process	10	Describe method of data extraction from reports (e.g., piloted forms, independently, in duplicate) and any processes for obtaining and confirming data from investigators.	3-4
Data items	11	List and define all variables for which data were sought (e.g., PICOS, funding sources) and any assumptions and simplifications made.	4
Risk of bias in individual studies	12	Describe methods used for assessing risk of bias of individual studies (including specification of whether this was done at the study or outcome level), and how this information is to be used in any data synthesis.	4

Summary measures	13	State the principal summary measures (e.g., risk ratio, difference in means).	4
Synthesis of results	14	Describe the methods of handling data and combining results of studies, if done, including measures of consistency (e.g., I^2) for each meta-analysis.	4
Risk of bias across studies	15	Specify any assessment of risk of bias that may affect the cumulative evidence (e.g., publication bias, selective reporting within studies).	4
Additional analyses	16	Describe methods of additional analyses (e.g., sensitivity or subgroup analyses, meta-regression), if done, indicating which were pre-specified.	4
**RESULTS**			
Study selection	17	Give numbers of studies screened, assessed for eligibility, and included in the review, with reasons for exclusions at each stage, ideally with a flow diagram.	3
Study characteristics	18	For each study, present characteristics for which data were extracted (e.g., study size, PICOS, follow-up period) and provide the citations.	Table 1
Risk of bias within studies	19	Present data on risk of bias of each study and, if available, any outcome level assessment (see item 12).	18; Table S2
Results of individual studies	20	For all outcomes considered (benefits or harms), present, for each study: (a) simple summary data for each intervention group (b) effect estimates and	Figure 2 and 3

		confidence intervals, ideally with a forest plot.	
Synthesis of results	21	Present results of each meta-analysis done, including confidence intervals and measures of consistency.	14-18; Figure 2 and 3
Risk of bias across studies	22	Present results of any assessment of risk of bias across studies (see Item 15).	18, Table S2
Additional analysis	23	Give results of additional analyses, if done (e.g., sensitivity or subgroup analyses, meta-regression [see Item 16]).	15-18; Figure 2 and 3
**DISCUSSION**			
Summary of evidence	24	Summarize the main findings including the strength of evidence for each main outcome; consider their relevance to key groups (e.g., healthcare providers, users, and policy makers).	19-21
Limitations	25	Discuss limitations at study and outcome level (e.g., risk of bias), and at review-level (e.g., incomplete retrieval of identified research, reporting bias).	19-21
Conclusions	26	Provide a general interpretation of the results in the context of other evidence, and implications for future research.	19-21
**FUNDING**			
Funding	27	Describe sources of funding for the systematic review and other support (e.g., supply of data); role of funders for the systematic review.	21

Figure S1. Preferred Reporting Items for Systematic Reviews and Meta-Analyses (PRISMA) checklist. *From:* Moher D, Liberati A, Tetzlaff J, Altman DG, The PRISMA Group (2009). Preferred Reporting Items for Systematic Reviews and Meta-Analyses: The PRISMA Statement. PLoS Med 6(7): e1000097. doi:10.1371/journal.pmed1000097
For more information, visit: www.prisma-statement.org.

Table S1. Mean age, proportion of female medical students and proportion of single medical students in sixty-nine studies examining anxiety in medical students.

First Author, Year	Mean Age, Years	Female Medical Students, Number	Female Medical Students,%	Single Medical Students, Number	Single Medical Students, %
Abdallah, 2014	18.02	197	52.0	375	98.9
Ahmed, 2009	NR	NR	NR	NR	NR
Akhtar, 2019	24.92	76	62.0	NR	NR
Akvardar, 2003	NR	175	39.1	NR	NR
Alaqeel, 2017	NR	NR	NR	NR	NR
Alvi, 2010	21.40	202	72.4	NR	NR
Amr, 2008	20.70	147	47.3	NR	NR
Ashton, 1995	20.40	109	58.6	NR	NR
Azad, 2017	20.60	112	74.6	NR	NR
Bassols, 2014	NR	NR	NR	NR	NR
Baykan, 2012	24.50	86	44.6	185	95.8
Bogowicz, 2018	NR	NR	NR	NR	NR
Casey, 2016	NR	62	51.0	NR	NR
Chandavarkar, 2007	NR	282	66.0	NR	NR
Chen, 2018	23.53	49	34.3	NR	NR
Coentre, 2016	23.25	304	66.7	422	92.5
de Sousa, 2018	21.69	336	65.6	498	97.3
Del-Ben, 2013	19.10	27	31.8	NR	NR
Ediz, 2017	21.99	458	49.4	NR	NR
El-Gilany, 2008 (Egypt)	20.60	0	0.0	NR	NR
El-Gilany, 2008 (Saudi Arabia)	21.00	0	0.0	NR	NR
Eller, 2006	21.30	318	77.0	NR	NR
Fawzy, 2017	21.22	452	64.6	688	98.3
Francis, 2019	21.18	403	64.8	507	81.5
Gan, 2019	23.00	85	57.0	147	98.7
Gaspersz, 2012	NR	NR	NR	NR	NR
Ghodasara, 2011	NR	147	49.0	262	87.0
Haghighi, 2018	22.04	101	49.0	174	84.0
Hu, 2019	NR	NR	NR	NR	NR
Ibrahim, 2013	21.10	450	100.0	422	93.8
Ibrahim, 2015	NR	82	50.0	NR	NR

Ibrayeva, 2018	NR	NR	NR	NR	NR
Iqbal, 2015	NR	NR	NR	NR	NR
Jafari, 2017	NR	NR	NR	NR	NR
James, 2017	23.91	264	42.4	509	81.7
Karaoglu, 2010	19.02	128	44.1	NR	NR
Knipe, 2018	NR	NR	NR	NR	NR
Kulsoom, 2015	NR	NR	NR	NR	NR
Kunwar, 2016	NR	NR	NR	NR	NR
Leao, 2011	24.60	63	44.0	NR	NR
Liu, 1997	18.20	NR	NR	NR	NR
Lupo, 2011	25.10	NR	NR	101	85.0
Mahroon, 2018	NR	194	63.2	NR	NR
Manchevska, 2014	NR	NR	NR	NR	NR
Mayer, 2016	22.80	714	52.9	NR	NR
Mousa, 2016	NR	NR	NR	NR	NR
Moutinho, 2017	22.10	425	55.8	NR	NR
Moutinho, 2019	21.00	200	64.1	NR	NR
Newbury-Birch, 2000	18.80	NR	NR	NR	NR
Nimkuntod, 2016	19.51	121	56.8	NR	NR
Pagnin, 2014	21.35	70	55.0	NR	NR
Pickard, 2000	NR	NR	NR	NR	NR
Prinz, 2012	NR	54	74.0	NR	NR
Rab, 2008	20.70	87	100.0	NR	NR
Rehmani, 2018	20.91	125	50.0	NR	NR
Rezaei, 2018	21.69	281	50.8	548	99.1
Samaranayake, 2011	NR	132	51.8	NR	NR
Saravanan, 2014	NR	181	50.6	NR	NR
Serra, 2015	22.70	402	61.2	642	97.7
Shi, 2015	21.65	1897	64.9	NR	NR
Sun, 2011	19.63	5455	53.8	NR	NR
Talih, 2018	NR	NR	NR	NR	NR
Tempski, 2015	22.80	714	52.9	NR	NR
van Venrooij, 2015	21.20	327	75.5	NR	NR
Villacura, 2017	22.20	136	49.1	NR	NR
Wahed, 2017	20.15	270	61.1	408	92.3
Waqas, 2015	19.90	NR	NR	NR	NR

Waqas, 2018	19.86	253	61.9	NR	NR
Wolf, 1998	24.00	50	44.0	NR	NR
Zhang, 2018	19.70	401	65.1	NR	NR

Abbreviations: NR, Not reported.

Table S2. Quality Appraisal & Ethical Approval of 30 studies examining prevalence of anxiety in medical students[a].

Number	First Author, Year	Selection (Maximum 5 Points)	Comparability (Maximum 2 Points)	Outcome (Maximum 3 Points)	Total Score	Ethical Approval
1	Abdallah, 2014	4	1	2	7	Y
2	Ahmed, 2009	4	1	2	7	N
3	Akhtar, 2019	4	2	2	8	Y
4	Akvardar, 2003	5	2	2	9	Y
5	Alaqeel, 2017	4	1	2	7	Y
6	Alvi, 2010	4	1	2	7	Y
7	Amr, 2008	5	2	2	9	Y
8	Ashton, 1995	4	2	2	8	N
9	Azad, 2017	4	1	2	8	N
10	Bassols, 2014	4	2	2	8	Y
11	Baykan, 2012	5	1	2	8	Y
12	Bogowicz, 2018	5	2	2	9	Y
13	Casey, 2016	4	1	2	7	Y
14	Chandavarkar, 2007	5	2	2	9	Y
15	Chen, 2018	5	2	2	9	Y
16	Coentre, 2016	5	1	2	8	Y
17	de Sousa, 2018	4	2	2	8	Y
18	Del-Ben, 2013	4	1	2	7	Y
19	Ediz, 2017	4	2	2	8	Y
20	El-Gilany, 2008 (Egypt)	4	2	2	8	Y
21	El-Gilany, 2008 (Saudi Arabia)	4	2	2	8	Y
22	Eller, 2006	5	2	2	9	Y
23	Fawzy, 2017	4	2	2	8	Y
24	Francis, 2019	5	2	2	9	Y
25	Gan, 2019	4	2	2	8	Y
26	Gaspersz, 2012	4	1	2	7	Y
27	Ghodasara, 2011	5	2	2	9	Y
28	Haghighi, 2018	5	2	2	9	Y
29	Hu, 2019	5	1	2	8	Y
30	Ibrahim, 2013	4	2	2	8	Y

31	Ibrahim, 2015	4	1	2	7	N
32	Ibrayeva, 2018	4	2	2	8	Y
33	Iqbal, 2015	4	2	2	8	Y
34	Jafari, 2017	4	1	2	7	Y
35	James, 2017	5	2	2	9	Y
36	Karaoglu, 2010	4	1	2	7	Y
37	Knipe, 2018	4	2	2	8	Y
38	Kulsoom, 2015	4	2	2	8	Y
39	Kunwar, 2016	4	1	2	7	Y
40	Leao, 2011	4	1	2	7	Y
41	Liu, 1997	5	2	2	9	N
42	Lupo, 2011	4	2	1	7	Y
43	Mahroon, 2018	5	2	2	9	Y
44	Manchevska, 2014	4	1	2	7	Y
45	Mayer, 2016	4	1	2	7	Y
46	Mousa, 2016	5	1	2	8	Y
47	Moutinho, 2017	5	1	2	8	Y
48	Moutinho, 2019	5	2	2	9	Y
49	Newbury-Birch, 2000	4	1	2	7	N
50	Nimkuntod, 2016	5	1	2	8	Y
51	Pagnin, 2014	5	2	2	9	Y
52	Pickard, 2000	5	2	2	9	N
53	Prinz, 2012	4	2	2	8	N
54	Rab, 2008	4	2	2	8	Y
55	Rezaei, 2018	5	2	2	9	Y
56	Samaranayake, 2011	4	2	2	8	Y
57	Saravanan, 2014	4	1	2	7	Y
58	Serra, 2015	5	1	2	8	Y
59	Shi, 2015	5	2	2	9	Y
60	Sun, 2011	5	2	2	9	Y
61	Talih, 2018	4	2	2	8	Y
62	Tempski, 2015	4	1	2	7	Y
63	van Venrooij, 2015	4	2	2	8	Y
64	Villacura, 2017	5	1	2	8	Y
65	Wahed, 2017	5	2	2	9	Y

66	Waqas, 2015	4	1	2	7	Y
67	Waqas, 2018	5	1	2	8	Y
68	Wolf, 1998	4	2	2	8	N
69	Zhang, 2018	4	2	2	8	Y

Abbreviations: Y, ethical approval provided; N, ethical approval not provided or not mentioned in article. [a] The adapted version of the Newcastle-Ottawa cohort scale for cross-sectional studies was used to appraise the quality of the sixty-nine studies. This adapted scale takes into consideration the selection of samples, comparability of subgroups and evaluation of outcome measures.

Table S3. Prevalence rate of anxiety stratified by gender as reported by twenty-four studies examining anxiety in medical students.

First Author, Year	Gender	Sample Size	Number of Medical Students with Anxiety	Prevalence of Anxiety, %
Alvi, 2010	Female	202	58	28.6
	Male	77	11	14.2
Amr, 2008	Female	147	86	58.8
	Male	164	63	38.5
Ashton, 1995	Female	109	33	30.0
	Male	77	12	16.0
Azad, 2017	Female	112	45	39.8
	Male	38	15	38.6
De Sousa, 2018	Female	336	39	11.6
	Male	176	14	7.9
El-Gilany, 2008 (Egypt)	Male	304	191	62.9
El-Gilany, 2008 (Saudi Arabia)	Male	284	155	54.6
Eller, 2006	Female	318	138	43.3
	Male	95	37	38.7
Ghodasara, 2011	Female	147	126	85.5
	Male	154	119	77.4
Ibrahim, 2015	Female	82	60	73.6
	Male	82	47	57.2
Ibrayeva, 2018	Female	634	286	45.1
	Male	844	350	41.4
Iqbal, 2015	Female	208	21	10.0
	Male	145	15	10.3
Jafari, 2017	Female	238	125	52.5
	Male	238	83	35.1
Karaoglu, 2010	Female	128	48	37.5
	Male	225	53	23.4
Kunwar, 2016	Female	280	44	15.8
	Male	258	31	12.0
Liu, 1997	Female	371	169	45.6
	Male	166	42	25.3
Mahroon, 2018	Female	187	82	43.7
	Male	120	47	38.8
Mayer, 2016	Female	714	111	15.5
	Male	636	151	23.8
Newbury-Birch, 2000	Female	130	25	18.9
	Male	64	16	24.7
Pickard, 2000	Female	90	11	12.4
	Male	46	20	43.0
Rab, 2008	Female	87	32	37.0
Sun, 2011	Female	5460	2129	39.0
	Male	4680	1357	29.0
Villacura, 2017	Female	136	17	12.5
	Male	141	18	12.7
Wahed, 2017	Female	270	116	43.1
	Male	172	58	33.8

Table S4. Prevalence rate of anxiety stratified by pre-clinical or clinical year of study reported by twenty-five studies examining anxiety in medical students.

First Author, Year	Year	Pre-Clinical or Clinical[a]	Sample Size	Number of Medical Students with Anxiety	Prevalence of Anxiety, %
Akvardar, 2003	M1	Pre-clinical	304	96	31.6
	Last year	Clinical	143	19	13.0
Alvi, 2010	M2	Pre-clinical	86	53	61.6
	M3, M4, M5	Clinical	193	36	41.5
Azad, 2017	M1,M2	Pre-clinical	134	21	15.7
	M3, M4, M5	Clinical	220	14	21.4
Bassols, 2014	M1	Pre-clinical	110	34	30.8
	M6	Clinical	122	11	9.4
Baykan, 2012	Last year	Clinical	193	97	50.3
Bogowicz, 2018	M1, M2	Pre-clinical	624	199	31.9
	M5	Clinical	265	114	43.0
Coentre, 2016	M4, M5	Clinical	456	90	19.8
Del-Ben, 2013	M1	Pre-clinical	85	16	18.8
Francis, 2019	M1, M2	Pre-clinical	249	31	12.4
	M3, M4, M5	Clinical	373	77	20.6
Gaspersz, 2012	M1-M4	Pre-clinical	814	260	32.0
	M5, M6	Clinical	316	88	28.0
Ibrahim, 2013	M2, M3	Pre-clinical	202	37	31.7
	M4, M5, M6	Clinical	248	39	37.5
Karaoglu, 2010	M1, M2	Pre-clinical	290	11	10.3
Leao, 2011	Last year	Clinical	144	39	27.0
Liu, 1997	M1, M2	Pre-clinical	498	61	12.3
	M3	Clinical	39	6	15.4
Manchevska, 2014	M1,M2	Pre-clinical	445	76	17.1
Nimkuntod, 2016	M1,M2,M3	Pre-clinical	213	16	25.8
Pagnin, 2014	M2	Pre-clinical	127	59	46.4
Prinz, 2012	M4,M5	Clinical	73	5	6.8

Rab, 2008 (female students only)	M1, M2	Pre-clinical	33	13	39.4
	M3, M4, M5	Clinical	54	25	46.3
Rezaei, 2018	M1,M2,M3	Pre-clinical	553	164	29.6
Saravanan, 2015	M1, M2	Pre-clinical	182	24	33.6
	M3,M4,M5	Clinical	176	29	44.3
Serra, 2015	M1,M2	Pre-clinical	160	37	23.1
	M3-M6	Clinical	497	104	20.9
Sun, 2011	M1, M2	Pre-clinical	10140	1430	14.1
van Venrooij, 2015	M1-M4	Pre-clinical	433	126	29.1
Wolf, 1998	M1	Pre-clinical	114	46	40.0

[a] Medical students are said to be in their pre-clinical years or clinical years as determined by their school curriculum.

Table S5. Comparison of prevalence rate of anxiety between medical students and non-medical students as reported by eight studies.

First Author, Year	Sample Size of Medical Students	Number of Medical Students with Anxiety	Prevalence of Anxiety, %	Comparison Group	Sample Size of Non-Medical Students	Number of Non-Medical Students with Anxiety	Prevalence of Anxiety, %
Bogowicz, 2018	889	313	35.2	Law students	353	155	43.9
de Sousa, 2018	512	121	23.6	Economics, finance and management students	240	40	16.8
Ibrahim, 2015	164	72	43.9	Pharmacy students	164	48	29.3
Knipe, 2018	583	145	24.9	Dentistry and Vet Science students	556	166	29.9
Lupo, 2011	119	35	29.4	Humanities and exact science students	51	26	51.0
Manchevska, 2014	445	76	17.1	Law and Dentistry Students	297	37	12.3
Prinz, 2012	73	5	6.85		109	30	27.5
Samaranayake, 2011	255	89	35.0	Nursing, health science, and architecture students	339	84	24.8
